# Nail lichen planus associated with imatinib mesylate

**DOI:** 10.1016/j.jdcr.2024.12.039

**Published:** 2025-01-31

**Authors:** Eden Axler, Eric Loesch, Toral Vaidya, Zachary Neubauer, Shari R. Lipner

**Affiliations:** a Department of Dermatology, Weill Cornell Medicine, New York, New York; b Department of Dermatology, Columbia University, New York, New York

**Keywords:** imatinib mesylate, lichenoid drug reaction, nail changes, nail lichen planus, nails

## Introduction

Imatinib, a tyrosine kinase inhibitor Food and Drug Administration (FDA) approved in 2001 for chronic myeloid leukemia (CML), is linked to adverse cutaneous reactions. Lichen planus is an inflammatory skin condition presenting as a papulosquamous eruption, with flat purplish papules.^1^ While imatinib-induced lichenoid drug eruption is uncommon, nail lichen planus (NLP) associated with imatinib is exceptionally rare, but may result in catastrophic nail damage.^1^^,^^2^ The growing use of imatinib underscores the importance of dermatologists promptly recognizing and managing these drug-induced adverse reactions to prevent irreversible nail dystrophy and loss. Herein, we present a case of NLP associated with imatinib therapy for CML.

## Case

A 75-year-old male, Fitzpatrick skin type II, with history of CML, presented with an 18-year history of nail changes. He had no other past medical history and was negative for human immunodeficiency virus, hepatitis B and C. He had been taking imatinib, 300 mg/d, for 18 years, which was discontinued 2 years before consultation due to CML remission. Shortly after initiating imatinib, the patient developed a lichenoid rash with mucocutaneous involvement and nail changes. Oral and skin lesions were managed with mouthwash (nystatin, hydrocortisone, and diphenhydramine), and topical steroids, respectively, with complete resolution after imatinib discontinuation. He reported difficulty in picking up objects and was embarrassed by the nail appearance.

**Fig 1:**
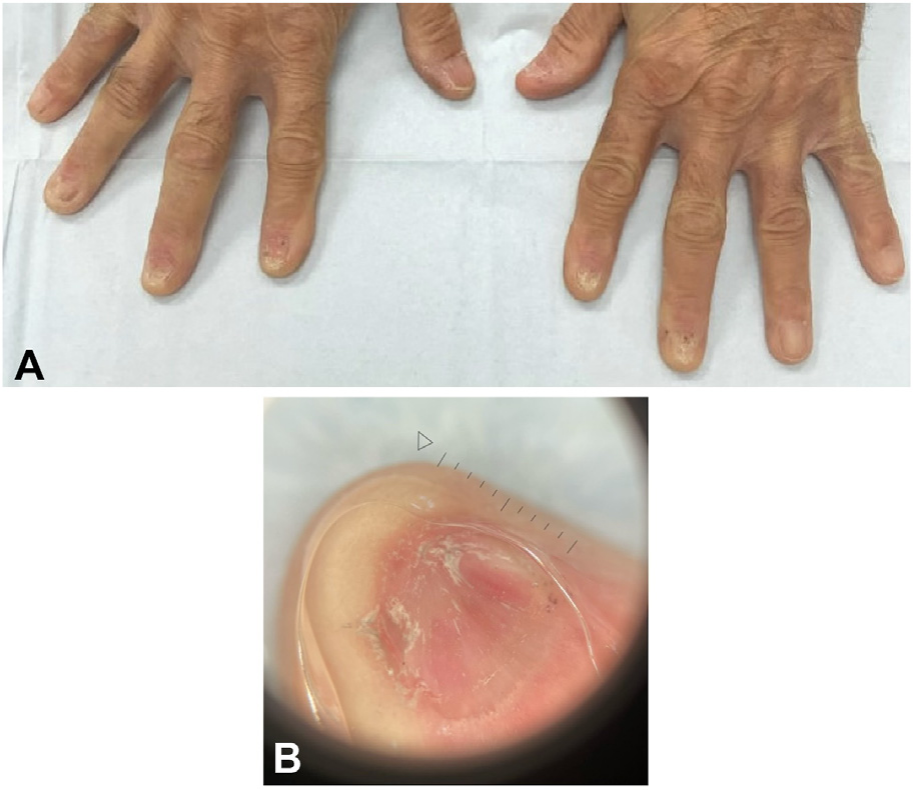
**A** and **B,** A 75-year-old male with nail lichen planus secondary to imatinib use. Fingernails atrophic with pterygium on 7/10 fingernails, sparing the right thumbnail and left fourth and fifth fingernails. On dermoscopy, severe nail plate thinning and atrophy is apparent.

Physical examination showed fingernail and toenail atrophy. Onyscopic evaluation showed fissuring, thinning, fragility, and pterygium (Fig 1). Considering the characteristic nail findings, history of imatinib use, and history of skin and oral mucocutaneous involvement, a diagnosis of NLP was favored. Since his nail findings were stable for 15 years, a nail biopsy was not recommended because it was unlikely to be conclusive or change management.

The patient was treated with 3 courses of intramatricial triamcinolone (2.5 mg/ml in 1% lidocaine, 0.5 cc) with only mild improvement.

## Discussion

We present a case of imatinib induced NLP presenting with onychodystrophy and almost complete nail loss. While the skin and oral mucosa findings resolved, nail changes progressed. Imatinib induced NLP has only been reported in 1 previous report. Following 5 months of 400 mg/d of imatinib, a 31-year-old male exhibited right second fingernail longitudinal ridging and widespread symmetrical, pruritic, violaceous papules, nodules, and plaques. No nail biopsy was performed, though a skin biopsy confirmed a diagnosis of lichen planus. Skin and nail changes resolved with topical steroids.^3^ In contrast to this case, our patient exhibited more severe, polydactylous onychodystrophy, which after 10 years of progression, did not resolve with treatment.

NLP is a progressive condition characterized by a longitudinal pattern of nail plate ridging and fissuring. If not promptly treated, may result in irreversible nail changes or permanent loss, significantly impacting daily life.^2^^,^^4^, ^5^, ^6^ Clinical features of disease vary depending on its severity, with pterygium and anonychia representing the most severe and irreversible manifestations.^4^ In a NLP consensus paper, clinical severity was categorized into mild, moderate, and severe. Mild cases are characterized by thinning, longitudinal ridging, distal splitting <3 mm in length, onycholysis <25%, and no hyperkeratosis in the nail bed. Moderate cases exhibit partial fissuring, longitudinal ridging, distal splitting between 3 and 5 mm, onycholysis between 25% and 50%, spotty erythema of the lunula, and subungual hyperkeratosis. Severe cases present with complete fissuring, deep longitudinal ridging, splitting >5 mm, onycholysis >50%, and diffuse erythema of the lunula.^2^ Approximately 20% of patients develop dorsal pterygium, 1.3% experience anonychia due to scarring, and severity may vary between nails.^7^

The diagnosis of nail NLP is predominately clinical, and a nail biopsy with histopathology may be helpful when the disease is active. For a patient undergoing chemotherapy who presents with suspected NLP, the initial workup includes inspecting the skin and oral mucosa for characteristic lichenoid changes. It is important to rule out other nail dystrophies that can present during chemotherapy. Onychomycosis and nail psoriasis can be ruled out histopathologically via nail clippings. The nail changes observed in this patient are characteristic of the progressively severe and refractory nail dystrophy that may occur with NLP. If the patient had presented 10 years earlier, aggressive early treatment may have prevented permanent onychodystrophy.

NLP treatment is challenging due to the lack of a specific molecular target for drug development. Moreover, it must be treated aggressively to prevent permanent nail damage. Based on an expert consensus, first-line treatment for NLP is intralesional steroids to the nail bed and matrix. The mechanism of chemotherapy induced NLP may be different from other NLP types and treatment outcomes may differ.^2^ Our patient had nail symptoms for over a decade without treatment, which likely resulted in the refractory and severe nature of his disease. If patients do not respond sufficiently to intralesional steroids, 2nd line treatment is systemic steroids administered orally or intramuscularly, but should be avoided in most oncology patients.^2^ Oral acitretin can be considered in these patients. Janus kinase inhibitors are theorized to target the Janus kinase signal transducer and activator of transcription protein pathway, believed to be integral to the inflammatory cascade underlying NLP pathology. While Janus kinase inhibitors have shown encouraging results in NLP case reports, they currently lack FDA approval for NLP.^8^

Nail changes induced by imatinib that are nonclinically characteristic of NLP, have been described, including melanonychia, nail plate thickening, and onycholysis (Table I). One case series describes 3 patients with CML treated with imatinib (one 300 mg/d, two 400 mg/d, average 8.6 weeks [range 4-12 weeks]) who developed psoriasiform nail changes and palmoplantar hyperkeratosis over several weeks.^9^ Imatinib induced blue nail discoloration has been reported in about 3% of cases, with possible mechanisms including the stimulation of melanocytes via c-KIT activation, accumulation of drug metabolites binding with iron or melanin, or medication induced immune dysregulation prompted by the medication, resulting in the excess release of melanin pigment.^10^

**Table I:** Summary of imatinib induced nail changes in the literature which do not meet clinical criteria for nail lichen planus

Age	Sex	Daily dose [mg]	Nail finding	Time to nail finding	Other affected sites	Reference
22	F	400	Blue discoloration	1 mo	None	9
47	M	400-800	Blue melanonychia	N/A	Pretibial, nose, posterior axillar folds, and hard palate pigmentation	11
40	M	N/A	Brown nail discoloration	4 mo	None	12
48	M	400	Transverse melanonychia	9 y	Oral mucosa pigmentation	13
53	M	400	Nail necrosis	4 mo	Grade 2 maculopapular rash of forearms and mouth	14
48	F	400	Hyperkeratosis of the toes	5 mo	Plantar lesions with yellow-brownish plaques and palmar desquamations	15
61	F	400	Nail thickening and subungual hyperkeratosis	8 wk	Erosive lesions and crusting of the lips, reticular white striations in oral mucosa pruritic, violaceous, flat-topped papules on her waist and buttocks	16
55	M	400	Pitting, onycholysis, subungual hyperkeratosis	4 mo	Exacerbation of psoriasis	17
24	F	400	Brown pigmentation involving all 20 nails	2 mo	N/A	18
86	M	400	Trachyonychia with onycholysis	3 mo	Pruritic rash, white streaks, and scaling of face, scalp, trunk, limbs	19
52, 68, 47	M, M, F	300, 400, 400	Onycholysis, hyperkeratosis of palms and soles, nail pitting	10 wk, 1 mo, 3 mo	Disseminated cutaneous eruption on his trunk, periorbital edema, scaly erythema	10
47	F	800	Onycholysis, subungual hyperkeratosis, and onychomadesis	N/A	Well-demarcated brightly erythematous and hyperkeratotic plaques on palms, soles, back, oral mucosa, labia	20
61	M	400	Discoloration and unspecified dystrophy	1 mo	Disseminated symmetrical, well-demarcated, violaceous, discrete plaques sparing the scalp	21
76	M	300 increased to 400	Subungual hyperkeratosis of fingernails and toenails	2 mo	Erythematous rash with lichenoid papules on the thorax and upper limbs	22

See Supplementary Text, available via Mendeley at https://data.mendeley.com/datasets/ffgydy6vzd/1 for table references.

## Conclusion

We present a case of lichenoid nail changes induced by imatinib, which may be an underrecognized finding. Vigilance is necessary regarding the potential for nail changes associated with imatinib administration. Collaboration with oncology and early intervention may mitigate NLP progression and prevent permanent nail damage.

## Conflicts of interest

None disclosed.
